# Motivational control of habits: A preregistered fMRI study

**DOI:** 10.1162/IMAG.a.100

**Published:** 2025-08-07

**Authors:** Andreas B. Eder, Vanessa Mitschke, Finja Leibold, David Dignath, Matthias Gamer

**Affiliations:** Institute for Psychology, JMU Würzburg, Würzburg, Germany; Department of Psychology, University of Göttingen, Göttingen, Germany; Department of Psychology, University of Tübingen, Tübingen, Germany

**Keywords:** outcome-selective Pavlovian-to-instrumental transfer, reward devaluation, habit, goal-directed action, expected value of control, anterior cingulate cortex function, functional neuroimaging

## Abstract

Habitual action is typically distinguished from goal-directed action by its insensitivity to changes in reward value. There is an ongoing discussion whether this insensitivity is an intrinsic design feature of habits or, rather, a function of the cognitive system that controls these action tendencies. Our preregistered study investigated this issue using functional magnetic resonance imaging of brain activity before and after a reward devaluation in an outcome-selective Pavlovian-to-instrumental transfer (PIT) paradigm. Based on the expected-value-of-control theory, it was hypothesized that neural activity of the dorsal anterior cingulate cortex (dACC) would increase during the presentations of Pavlovian cues associated with a devalued outcome, reflecting increased control allocation in situations predictive of a devalued reward. The behavioral results confirmed an outcome-selective PIT effect that was abolished by devaluing the associated outcome. Contrary to our hypothesis, neuroimaging data revealed that dACC activity decreased during presentations of the associated cue. A comparable reduction was also observed in the ventromedial prefrontal cortex and the putamen. These findings suggest that the current reward value was accessed during the transfer tests and that devaluation of the action outcome did not enhance cognitive control over associated response tendencies. The study plan and data analyses were peer-reviewed prior to data collection by Peer Community In: Registered Reports (PCI:RR). The Preregistered Stage 1 protocol is available at https://osf.io/k8ygb (date of in-principle acceptance: February 8, 2022; https://rr.peercommunityin.org/articles/rec?id=140). The Stage 2 report was recommended after peer review by PCI:RR at https://doi.org/10.24072/pci.rr.101079 (date of recommendation: July 8, 2025).

## 1 Introduction

A hallmark of habitual actions is that, once they are established, they become insensitive to changes in the values of associated action rewards. In dual action psychology, *habitual* actions are defined as behaviors that are “simply triggered by the appropriate stimulus”, which are contrasted with *goal-directed* actions that are controlled “by the current value of their goals through knowledge about the instrumental actions and their consequences” (Dickinson, 1985, p. 67). This distinction is also propagated by neuroscientific models that distinguished between *model-based* (goal-directed) and *model-free* (habitual) action control modes, and both are subserved by distinct but interacting neural systems (Daw et al., 2005). Model-based action control has been proposed to depend on an internal model of the world that explicitly relates alternative actions to future environmental states. This mode implicates regions of the prefrontal cortex and their connections to dorsomedial striatum (caudate nucleus in primates). By contrast, model-free control relies on the retrieval of cached action values from memory without requiring an elaborate mental model to be constructed or searched; this mode is implemented by a sensorimotor cortex–basal ganglia loop that includes the dorsolateral striatum (putamen in primates) (for reviews see Balleine & O’Doherty, 2010; Dolan & Dayan, 2013; Graybiel & Grafton, 2015; Yin & Knowlton, 2006).

Notably, the mode of behavior control could be determined with experimental outcome revaluation procedures that change the value of associated action outcomes after training and examine the effect on behavioral performance: If performance is sensitive to manipulations of outcome value (e.g., if the rate of responding decreases after outcome devaluation), then it is concluded that the behavior was controlled by the anticipation of the outcome—and hence goal-directed. If performance is insensitive to these manipulations, then it is concluded that the behavior was controlled by antecedent stimuli—and hence habitual. Importantly, this test should occur in the absence of the revalued outcome to prevent new action learning during the (extinction) test.

A large number of behavioral and neuroscientific studies with rodents and humans were conducted using this revaluation assay. In an early study, Adams and Dickinson (1981) found that rats trained to press a lever for food would subsequently cease lever pressing in an extinction test after the food pellets were separately paired with a toxin (thereby devaluing the food reinforcer). They concluded that lever pressing was goal-directed. However, when the rats received more extended training with the food reinforcer, they continued to press the lever even after the devaluation treatment, demonstrating outcome insensitivity more consistent with a habitual control mode (Adams, 1982). Similar effects have been reported in fMRI studies with humans, demonstrating that orbitofrontal cortex (OFC) and amygdala regions track changes in the value of predicted rewards (Gottfried et al., 2003; O’Doherty et al., 2000; Tanaka et al., 2008; Valentin et al., 2007). With overtraining, however, cue-related activity in a specific region of the posterior dorsolateral putamen increased as the instrumental training progressed, which was interpreted as a shift from goal-directed to habitual action control (Tricomi et al., 2009).

To summarize, brain imaging studies on habit acquisition through overtraining found that the dorsolateral striatum is involved in habit acquisition, whereas the ventromedial prefrontal cortex is sensitive to changes in outcomes values and implicated in the control of goal-directed action. A logical problem with the overtraining procedure, however, is that it conflates the acquisition of habits with performance improvements that come with practice (i.e., the expression of acquired habits) (for a discussion of this issue, see Liljeholm et al., 2015). Therefore, the most stringent way for studying the implementation of habitual control is to exclude exposure to repeated S-R pairings before the test phase at all. This could be realized with an experimental paradigm that was dubbed “Pavlovian-to-instrumental transfer”—or in short: PIT.

## 2 Pavlovian-to-instrumental Transfer

**Fig. 1.**
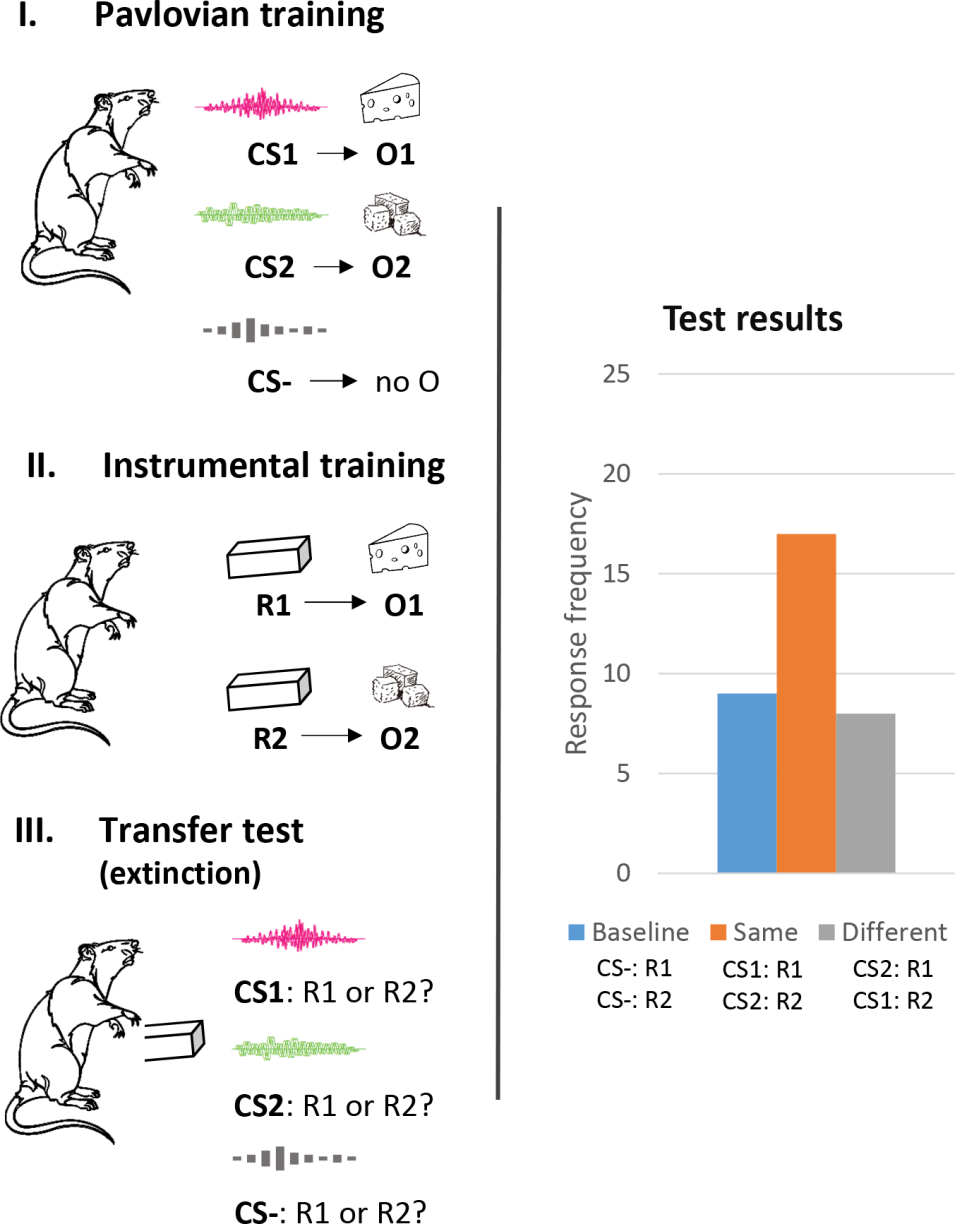
Illustration of the outcome-selective PIT paradigm with the hypothetical test result.

In outcome-selective PIT, reward-related cues that are predictive of particular rewards prime instrumental responses that are associated with these rewards. Figure 1 shows the basic procedure of an animal study using the PIT paradigm. In a first *Pavlovian training phase*, the animals learn to associate stimulus cues (e.g., CS1: a high-pitched tone and CS2: a low-pitched tone) with the delivery of particular outcomes (e.g., O1: food pellets and O2: a sucrose solution). In a subsequent *instrumental training phase*, they could earn the rewards by own responding (e.g., R1: lever on the left; R2: lever on the right). In a final *transfer test phase*, the animals could continue working for the rewards but this time with intermittent presentations of the reward-related cues and without delivery of the rewards (extinction test). Many studies with rodents and humans showed that the rate of responding for a specific reward is elevated by the presentation of a cue that is associated with the same reward (e.g., CS1: R1 > R2) relative to control conditions with an unpaired stimulus cue or baseline periods with no cue (see the hypothetical test result in the right panel of Fig. 1) (for reviews see Cartoni et al., 2016; Holmes et al., 2010). This effect was dubbed the outcome-selective PIT effect, or in short: the *specific* PIT effect.

Researchers also observed another transfer effect which was labeled *general* PIT effect. Here, the CS enhances responses directed to other rewards relative to control conditions in which no Pavlovian cue is present (Estes, 1943; Talmi et al., 2008). Furthermore, both types of effects could be investigated in a single paradigm which was named “a full transfer paradigm” (Cartoni et al., 2016).

The critical feature of the PIT paradigm is that Pavlovian relations and instrumental relations are trained in separate phases, which means that transfer in the test phase occurs without prior training of the instrumental action in the presence of Pavlovian cues. This design, therefore, allows to study the expression of habits distinct from habit acquisition.

Researchers proposed different accounts for specific and general PIT effects. General PIT effects were typically explained with CS-triggered activations of motivational systems on a central level that prime preparatory responses for appetition and defense (Dickinson & Balleine, 2002; Rescorla & Solomon, 1967). Specific PIT effects, in contrast, were typically explained with a cue-triggered activation of sensory representations of action outcomes that, in turn, primes the response producing that outcome (S-O-R theory; Trapold & Overmier, 1972). These theories assume that PIT effects are mediated by a chain of associative links that form during the training phases. Modern accounts of specific PIT effects in humans also highlighted the role of propositional processes and the influence of a person’s explicit beliefs about the availability of outcomes and their values (Mahlberg et al., 2021). This propositional approach was also supported by studies of task instructions that could reverse the direction of specific PIT effects without prior training, but only if the person had sufficient processing resources (Seabrooke et al., 2016; Seabrooke, Wills, et al., 2019).

Neuroscientific studies with animals have shown that a distributed set of brain regions is necessary for the expression of PIT, including the amygdala (Blundell et al., 2001; Hall et al., 2001; Holland & Gallagher, 2003), nucleus accumbens (Hall et al., 2001; Corbit et al., 2001; de Borchgrave et al., 2002), ventral striatum (Cardinal et al., 2003; Corbit & Balleine, 2005), and ventral tegmental area (Corbit et al., 2007; Murschall & Hauber, 2006), suggesting the involvement of dopaminergic pathways (Lex & Hauber, 2008). Lesions studies in rats found that specific transfer was abolished by basolateral amygdala and nucleus accumbens shell lesions, whereas general transfer was abolished by lesions of the central nucleus of the amygdala and the nucleus accumbens core (Corbit & Balleine, 2005, 2011). This is corroborated by human neuroimaging studies that reported an involvement of the striatum in specific PIT (Bray et al., 2008; Lewis et al., 2013; Mendelsohn et al., 2014; van Steenbergen et al., 2017; van Timmeren et al., 2020) and of the amygdala and nucleus accumbens in general PIT (Prévost et al., 2012; Talmi et al., 2008). In neuroimaging analyses of specific transfer effects, Bray et al. (2008) reported a contribution of the ventrolateral putamen but not the amygdala in specific PIT. Using high-resolution brain-imaging, Prévost et al. (2012), however, could show that, in addition to the ventrolateral putamen, a region in the ventral amygdala within the boundaries of the basolateral (BLA) complex is involved, which accords with lesion studies on rodents (Johnson et al., 2009). The BLA is suggested to be involved in the processing of specific sensory features of an outcome (Balleine & Killcross, 2006), which, in turn, may affect action selection and execution via the acquired O-R link.

We know of only one published fMRI study with humans that investigated neuronal correlates of specific transfer effects following the devaluation of an action outcome (van Steenbergen et al., 2017). In this study, participants were trained to associate specific keypresses and symbols with particular food rewards (popcorn, Smarties). Following the training, one of the two food rewards was devalued by feeding to satiety. Subsequently, participants could work again for the food rewards with intermittent presentations of the reward-related cues. Behavioral data showed that satiation failed to reduce cue-dependent food-seeking. Furthermore, during cued trials, the blood oxygenation level-dependent (BOLD) activity in a region of the putamen differentiated between actions that were consistent and inconsistent with the cued outcome. When choices were made in the absence of Pavlovian cues, the posterior ventromedial prefrontal cortex tracked the values of the expected food rewards. Overall, these findings accord with previous studies that suggested an involvement of the ventromedial prefrontal cortex (vmPFC) in goal-directed action (Gläscher et al., 2009; Gottfried et al., 2003; Valentin et al., 2007), and the putamen and BLA in cue-driven, habitual responding (Bray et al., 2008; Prévost et al., 2012).

## 3 Dual Action or Controlled Action?

Observations that general action preferences in the absence of cues were sensitive to post-training devaluation of outcomes, whereas action choice after cueing were not, were interpreted in support of a dual controller theory that explains specific transfer effects with operations of a ‘habit controlling system’ that functions autonomously from a value-based, goal-directed action system (e.g., Ceceli & Tricomi, 2018; de Wit et al., 2009; Hogarth, 2012; van Steenbergen et al., 2017). This interpretation was, however, challenged by an increasing number of studies that observed behavioral adjustments in various PIT tests following the devaluation of a reward (Eder & Dignath, 2016a, 2016b; Hinojosa-Aguayo & González, 2020; Seabrooke et al., 2017; Seabrooke, Hogarth, et al., 2019). A pioneering study (Allman et al., 2010) trained human participants to associate specific symbols and actions with particular monetary outcomes in separate learning phases. In a subsequent transfer test, participants exhibited a specific transfer effect. After this test, participants were informed that one of the currencies has lost its value due to a financial crash. Participants then worked again on a transfer test. Results showed that the cue associated with the now-devalued outcome has now lost its capacity to elevate the response rate. Eder and Dignath (2016a) reproduced this result using a similar paradigm and in another study when a food outcome (lemonade) was devalued by pairing it with bad-tasting Tween20. In this latter study, however, the elimination was observed only when participants were to drink the devalued lemonade immediately after the transfer phase but not when consumption was postponed. On the basis of these results, Eder and Dignath (2019) proposed that cue-motivated action tendencies in PIT tasks are not insensitive to outcome values, but, rather, by lacking the motivation for control. This motivation can be created (among other factors) by strong devaluation treatments that result in a complete and immediate loss of the reward (e.g., no monetary value), in comparison to standard treatments involving feeding to satiety, such as smoking cigarettes or eating chocolate, that likely leave substantial parts of the reinforcers intact (for a review see Kruglanski & Szumowska, 2020).

It should be highlighted that weak and/or incomplete outcome devaluation is not the only explanation for spared PIT tendencies in previous studies with humans. Other possible explanations are (i) species-specific processes differing between humans and rodents (de Wit et al., 2018; but see also Balleine & O’Doherty, 2010); (ii) systematic differences in baseline responding (Seabrooke, Hogarth, et al., 2019), (iii) residual beliefs about the informativeness of the Pavlovian cues with respect to the availability of outcomes (Seabrooke et al., 2017), (iv) and the operation of additional goals during the PIT test (De Houwer et al., 2018). Latter explanations concur in the present argument that the motivational insensitivity observed in human PIT studies was the result of a goal-dependent process—and not a feature of a ‘habitual action controller’ that is blind to reward expectations. This alternative approach to habit research explains cue-dependent action tendencies in PIT with a unitary account (Hommel, 2019; Kruglanski & Szumowska, 2020), and it questions the utility of goal-independency as exclusive criterion for the definition of ‘habitual’ behaviors (De Houwer, 2019).

Eder and Dignath (2019) referred to Expected Value of Control (EVC) theory for an account of the conditions in which cue-dependent PIT tendencies become motivationally controlled. The EVC theory assumes that (habitual) ‘default’ processes become cognitively controlled when the expected benefits of response control outweigh the intrinsic cost to engaging in control (Shenhav et al., 2013). According to this theory, a central hub for this weighting process is the dorsal anterior cingulate cortex (dACC) that receives inputs from brain areas responsible for the valuation of incoming stimuli or action outcomes (OFC, vmPFC, amygdala) and that sends output signals to areas responsible for the implementation of control (lateral PFC, motor cortex, striatum, subthalamic nucleus). It is assumed that the dACC monitors ongoing processing of signals indicative of the need for control, evaluates the demands for control, and allocates control to downstream regions (Botvinick, 2007; Shenhav et al., 2016). In PIT tasks, the default response that must be potentially overcome is the cue-instigated action tendency. Before the revaluation treatment, there exists no valuable action that could be selectively increased for a better payoff. Expected payoffs, however, change dramatically after a strong devaluation of the outcome. Now, there exists a clear difference in the value of action outcomes. Control is intensified when the costs of obtaining a devalued outcome justify the costs of engaging in control, resulting in a selective suppression of the associated response tendency. On the neural level, activity of the dACC in a transfer test should thus increase during presentations of Pavlovian cues predictive of the devalued outcome, indexing the monitoring and implementation of control. This hypothesis is tested in the proposed fMRI study.

## 4 Experiment

We used a stock market paradigm adapted from Allman et al. (2010) and Eder and Dignath (2016b, Experiment 2). Participants were first trained in separate phases to associate specific company emblems and instrumental actions with particular (fictitious) African currencies. Outcome-selective transfer in a PIT test was then measured by the extent to which a company emblem increased responses associated with the same currency. Then, one of the currencies was devalued by instruction, and cue-motivated response tendencies were again assessed in a second transfer test. This design, hence, allowed a comparison of specific PIT tendencies before and after the devaluation of a specific outcome.

The functionality of the design and experimental procedure was assessed in a pilot study (*n* = 24) conducted without functional imaging. A report of the pilot study is available at https://osf.io/79gew/.

### 4.1 Methods

#### 4.1.1 Participants

Sample size calculation was based on a-priori power analyses for statistical hypotheses central to the research question. Standardized effect sizes of behavioral effects obtained in the pilot study were used as effect size estimates for the power analyses. For this power analysis approach, we assumed a close relationship between magnitudes of behavioral effects and magnitudes of fMRI activity in brain regions hypothesized to mediate the behavioral effects (Eder & Dignath, 2019). As summarized in Table S1 in the Supplementary Information, four statistical effects are of particular theoretical relevance for the present research question. For our primary hypothesis test (H1), we planned sample size to have excellent power (1-β = 0.95) to detect *d_z_* ≥ 0.52 in a one-tailed paired t-test. The effect size estimate was obtained from our pilot study (dz = 0.55) and from a previous study (Eder & Dignath, 2016b, Exp 2, dz = 0.53) using the same procedure. For the manipulation checks (H2–H4), we selected *d_z_* = 0.40 as the smallest effect size of interest. We planned to use equivalence tests for a statistical assessment whether a manipulation check has failed (Lakens et al., 2018). Sample size was selected to have sufficient statistical power for the detection of this effect size in a one-tailed paired t-test.

We preregistered a data collection plan with a target of *N* = 41 participants and intended to replace dropouts until this number was reached. Four participants were replaced due to large head movements during MRI measurement (>2 mm translation or > 2° rotation within one of the Transfer Test phases). No replacements were necessary based on our other exclusion criteria (i.e., participants terminating the experiment or being unable to indicate the correct Pavlovian relation after one testing hour and/or the instrumental relations after relearning).

The final sample comprised 29 females and 12 males, with a mean age of 25.02 years (*SD* = 5.20, range = 18–39 years). Only right-handed individuals were recruited. All participants provided written informed consent. The study procedure was approved by the Institutional Review Board of the Institute for Psychology at the University of Würzburg (GZEK 2022-14).

#### 4.1.2 Design

The experiment had a 2 (transfer test: before devaluation vs*.* after devaluation) x 4 (Pavlovian relation: CS1/Currency 1 vs*.* CS2/Currency 2 vs*.* CS3/Currency 3 vs*.* CS−/no currency) x 3 (instrumental relation: R1/Currency 1 vs. R2/Currency 2 vs R3/Currency 3) repeated-measures design. R1 always worked for the (to-be) devalued currency. The following factors were counterbalanced across participants: (1) The Pavlovian assignment of the geometric figures (CS) to the outcomes using a Latin square; (2) the instrumental assignment of the money currencies to the three response buttons. This counterbalancing procedure resulted in 4 x 6 = 24 combinations.

#### 4.1.3 Apparatus and material

Data were acquired on a Siemens Skyra 3T scanner using a 32-channel head coil. Participants held a 5-button fiber optic response pad in their right hand, with a button on the left side (pressed with the thumb) and four buttons ergonomically arranged in a curve for the other fingers (Current Designs, Philadelphia, USA). The experiment was programmed using the software E-Prime 3.0 Professional with the EEfMRI extension (Psychology Software Tools, Inc.).

Pavlovian cues were 4 visually distinct geometric figures (1 star, 1 square, 1 triangle, 1 circle). Outcomes in the training phases were currency symbols: ‘B$’ for Botsuana Dollar; ‘N$’ for Niger Dollar; ‘T$’ for Tansania Dollar; and ‘–’ for no trade outcome.

#### 4.1.4 Procedure

Table 1 gives an overview of the experimental procedure that was adapted from Eder and Dignath (2016, Experiment 2). Participants read a vignette describing the participant in the role of a stockbroker. Companies in different African countries would trade with particular fictitious African currencies (B$, N$, T$). Participants’ tasks for the training phases were to figure out which company trades with which African currency (Pavlovian training) and to earn as many African dollars as possible (instrumental training). Instructions also highlighted that their profit in African dollars would be exchanged into real money (Euros) after the experiment.

**Table 1.** Summary of experimental procedure.

Stage 1	Stage 2	Stage 3	Stage 4	Stage 5	Stage 6	Stage 7	Stage 8
Exchange rates	Pavlovian training	Instrumental training	Transfer Test 1	Pavlovian retraining	Instrumental retraining	Revaluation	Transfer Test 2
50N$: €1	S1→ N$	R1→ N$	S1: R?	S1→ N$	R1→ N$	20 N$: €0	S1: R?
50 B$: €1	S2→ B$	R2→ B$	S2: R?	S2→ B$	R2→ B$	20 B$: €1	S2: R?
50 T$: €1	S3→ T$	R3→ T$	S3: R?	S3→ T$	R3→ T$	20 T$: €1	S3: R?
	S4→ –		S4: R?	S4→ –			S4: R?

*Note.* Conditioned stimuli (CS) were four geometrical shapes; instrumental responses (R) were pressing buttons on the response pad; and outcomes were symbols indicating earnings in different African dollar currencies (B$, N$, T$) or no earning (–). Exchange rates in Euros were displayed at the start of the experiment (Stage 1) and during the revaluation phase (Stage 7). The assignment of the outcomes to the geometric figures and to the buttons was counterbalanced across participants (for details see the Design section).

**Stage 1: Exchange Rates and Currency Rating.** The exchange rates of the African currencies were displayed on the screen, with 50 Dollars of any African currency equivalent to 1 Euro. Participants were then asked to evaluate each currency on a scale ranging from 1 (very bad) to 5 (very good).

**Stage 2: Pavlovian Training.** Participants were informed that geometric figures will appear on the screen that represent companies. The logo of one company was represented by a circle, a second company by a triangle, a third company by a square, and a fourth logo by a star. Instructions given for this phase were the following (translated from German):

In this part of the experiment, you will see various geometric symbols. Each symbol represents a company (company logo). One company has a CIRCLE as logo, another company a TRIANGLE, the third a SQUARE, and the fourth a STAR. Since the companies only trade in a single country, each company trades with exactly one currency.

FIND OUT WHICH COMPANY (Circle, Star, etc.) TRADES WITH WHICH SPECIFIC CURRENCY (B$, N$, etc.)!

Participants then observed 10 pairings of a company symbol with a trade outcome, distributed across 10 blocks with random presentations of each figure-currency pair in a block. The company symbol was presented for 1,000 ms; after an additional 50 ms, a currency symbol (e.g., 1B$) was presented as outcome for 2,000 ms. Participants were asked to press the thumb button during the presentation of a currency symbol to confirm the trade, and to refrain from pressing the button if the outcome was no trade (symbol: —). This task procedure was implemented to direct the participants’ attention to the outcomes. An error message appeared for 5,000 ms if the thumb button was not pressed within 2,000 ms following the presentation of the outcome or if the key was pressed in a trial with no trade outcome. The intertrial interval (ITI) ranged between 500 and 1,500 ms.

After training, participants were asked to indicate the contingencies between the companies and the currencies. In each trial, a company symbol (circle, star, etc.) was presented and four possible associates (the three currencies and ‘–’ indicating no outcome) below the company symbol. Participants indicated the paired outcome by pressing designated buttons. Each company symbol was presented once and in randomized order. A message after each button press indicated whether the participant’s assignment was correct or incorrect. If one or more assignments were incorrect, the Pavlovian training was repeated with a reduced number of training trials (4 x 5 trials). After retraining, the participant answered an additional Pavlovian contingency test, and the retraining continued until all answers were correct.

**Stage 3: Instrumental Training.** Participants were informed that they could now earn African dollars by pressing buttons on the response pad. Instructions stated that they could switch between the response buttons as often as they wished, though the computer might instruct them to stop pressing a particular button. In such cases, they had to use the remaining buttons to earn dollars in other currencies.

A black fixation cross was presented on a white background while participants responded on three concurrent fixed ratio nine schedules (FR 9). Participants responded by pressing three buttons on the response pad using their index, middle, and ring fingers, respectively. One response button worked for Botsuana dollars (B$), one for Niger dollars (N$), and the third for Tansania dollars (T$). Participants could switch between buttons at any time, and if they did so before reaching the FR9 criterion, they could resume completing the requirement for that button when they returned to it. Once a button had been pressed nine times, a dollar sign in an African currency (+1B$, +1N$, or +1T$) appeared for 2,000 ms. Participants were instructed to press the thumb button during this time to ‘bank’ the dollar to their account. Failure to press the thumb button resulted in an error message, and the dollar was not credited to their account. The computer prompted participants to stop responding on a particular button once they had earned 20 dollars in that currency (i.e., after 180 presses).

After instrumental training, participants indicated the correct instrumental contingency by pressing the button associated with the African dollar displayed on the screen. The African currencies were presented in a randomized order. If a participant made an incorrect selection, the instrumental training was repeated with half the number of outcome presentations (i.e., earning 10 dollars of each currency, requiring 90 presses per key).

**Stage 4: Transfer Test 1.** Instructions stated that they could continue earning African dollars through button presses, but their earnings would not be displayed in this phase. A dollar sign at the center of the screen signaled the time window for response registration: a green dollar sign indicated that the ‘stock market’ was open, while a red sign indicated it was closed. Button presses were only registered during the market’s open phase. In addition, the following information was given (translated from German):

From time to time, a company will also trade on the stock market (i.e., a company logo will appear on the screen). These company trades do not influence your own profits.

NOTE: Your profit in African dollars is not shown in this phase. A press of the thumb button is therefore not necessary.

The dollar sign remained green for 12,000 ms and then turned red for an additional 4,000–12,000 ms, following a positively skewed distribution (4–8 s in 75% and 9–12 s in 25% of the trials). Two seconds after onset of the green dollar sign, a company symbol (geometric figure) superimposed on the green dollar sign was shown for 8,000 ms. Each of the four company symbols presented during the Pavlovian phase was shown in randomized order in a block. The transfer test had 12 blocks (48 trials). With registration of the ninth press of a response key, one dollar was added to the tally of that African currency; however, profits in African dollars were not presented as outcomes during this stage, corresponding formally with an extinction test.

**Stages 5 to 8: Pavlovian and Instrumental Retraining, Outcome Devaluation, and Transfer Test 2.** Before devaluation, the Pavlovian training (Stage 4) and the instrumental training (Stage 5) were repeated with half the number of trials in each stage. This re-training served to reestablish the Pavlovian and instrumental contingencies after the extinction test (Transfer Test 1). Following retraining, and immediately prior to the revaluation treatment, the African dollars earned so far were exchanged for Euros, and the tally for each currency was reset to zero. Then, one of the currencies was devalued with the following instructions written with red font color (translated from German):

    — ALERT– — ALERT– — ALERT– —

        NEW EXCHANGE RATES!

The exchange rates of African dollars to Euros have changed due to an international financial crisis.

        Exchange rates are now:

             20N$ = €0

              20 T$ = €1

             20 B$ = €1

The devaluation of the currency was counterbalanced across participants. Participants worked through a second transfer test (Stage 7) that was identical with the first transfer test. Participants then rated again each currency as a manipulation check of the revaluation treatment (see Stage 1 for the rating procedure).

Finally, participants were paid and debriefed with respect to the nature of the study.

#### 4.1.5 MRI data acquisition and preprocessing

Functional data were obtained using a T2*-sensitive gradient echo-planar imaging (EPI) multiband sequence with 42 slices (voxel size 2 × 2 × 2 mm^3^, 1 mm gap between slices) oriented along the anterior commissure–posterior commissure axis (repetition time [TR] = 1,340 ms; echo time [TE] = 25 ms; flip angle = 60°; FOV = 216 × 216 mm; GRAPPA with PAT-factor 2; multiband acceleration factor 2). Additionally, isotropic high-resolution (1 × 1 × 1 mm^3^) structural images were recorded using a T1-weighted coronal-oriented MPRAGE sequence with 240 slices.

Image processing and statistical analyses were carried out using Statistical Parametric Mapping (SPM12; Wellcome Department of Imaging Neuroscience, London, UK). Unless otherwise noted, SPM12 default values were used for the respective preprocessing steps. We discarded the first four volumes of each time series to account for T1 equilibration effects. Volumes were slice time corrected, realigned, and unwarped using the mean EPI image. The structural T1 image was co-registered to the mean EPI image, and T1 images were segmented using the DARTEL procedure to create structural templates across subjects as well as individual flow fields. These flow fields were used to spatially normalize the EPI images as well as the structural T1 images into MNI space. For the EPI images, data were stored with a voxel size of 2 × 2 × 2 mm^3^ and smoothed using a 6 mm full-width at half maximum (FWHM) isotropic Gaussian kernel. Structural T1 images were stored with a voxel size of 1 × 1 × 1 mm^3^ and averaged across participants. The resulting mean image was used to illustrate the results of the functional analyses (i.e., overlay of statistical parametric maps).

#### 4.1.6 Analyses of behavioral data

Data were analyzed using IBM SPSS Statistics 27. Mean frequencies of button presses during the presentation of the CS (8 s) in Transfer Test 1 were analyzed using a 4 x 3 repeated-measures analysis of variance (rm-ANOVA) with the factors *Cue* (CS1, CS2, CS3, CS−) and *Response* (R1, R2, R3). It was hypothesized that response rates would be elevated by presentations of conditioned cues with common outcomes (i.e., a statistical interaction effect between Pavlovian Cue and Response). Outcome-selective PIT was assessed with a comparison of the response rate in the presence of cues associated with the same outcome relative to the response rate in the baseline condition with CS−. For follow-up comparisons, response rates in the presence of matching Pavlovian cues were consequently directly compared with those in the presence of neutral cues (i.e., CS1:R1 vs*.* CS−:R1, CS2:R2 vs*.* CS−:R2, CS3:R3 vs*.* CS−:R3) using paired-samples t-tests. For the second transfer test, response rates were analogously analyzed, whereby O1 designates the devalued currency.

Magnitudes of PIT effects in the two transfer tests were also directly compared to examine whether they were changed by the revaluation treatment. For this comparison, we first transformed the raw values into z scores to adjust for differences in the base rates of button presses after the devaluation treatment (Bush et al., 1993). The a priori significance level was set to α = .05 for all analyses, and *p*-values were corrected for violations of sphericity using Greenhouse-Geisser. Standardized effect sizes (Cohen’s *d,* partial eta-square) are reported where appropriate.

#### 4.1.7 Analyses of fMRI data

Data were analyzed using SPM12 (v7771) and Matlab 9.3.0.713579 (R2017b). A two-level random-effects GLM approach as implemented in SPM12 was used. At the single-subject level, we modeled the 8,000 ms response period of the Transfer Test 1 and Transfer Test 2 phases (i.e., when the dollar sign was green and a company symbol was shown) as separate boxcar functions for each *Pavlovian cue* (CS1, CS2, CS3, CS−). Additional regressors of no interest included boxcar functions for the initial 2,000 ms response phase without any *Pavlovian cue* and the 2,000 ms response phase after the offset of the *Pavlovian cue*, respectively. Individual behavioral responses were modeled as stick functions for each button press, separately for the different *Responses* (R1, R2, R3).^*^ All regressors were convolved with the canonical hemodynamic response function as implemented in SPM12. EPI images were high-pass filtered at 128 s, and an autoregressive AR(1) model was used to account for serial correlations in fMRI time series.

Statistical analyses on the group level involved one-sample t-tests on activation difference maps of each Pavlovian cue minus the neutral cue (i.e., CS1 vs*.* CS−, CS2 vs*.* CS−, CS3 vs*.* CS−) separately for each Transfer Test phase. Moreover, to directly compare patterns of brain activation before and after devaluation, we first assessed general differences between Transfer Test phases by calculating a paired t-test between average activation differences of all Pavlovian cues minus the neutral cue (i.e., (CS1 + CS2 + CS3)/3 vs*.* CS−) in the second as compared to the first Transfer Test phase. Afterward, we specifically focused on differences between devaluated and non-devaluated cues by calculating separate paired t-tests between activation difference maps of each Pavlovian cue minus the neutral cue (i.e., CS1 vs*.* CS−, CS2 vs*.* CS−, CS3 vs*.* CS−) in the second as compared to the first Transfer Test phase.

Analyses primarily focused on the dACC and we, therefore, used a small volume correction for this region of interest (ROI). Following previous suggestions (Spunt et al., 2012), the respective ROI was generated by extracting a binary mask including the bilateral anterior and middle cingulate and paracingulate gyri from the automated anatomical labeling atlas (Rolls et al., 2015) and trimming this mask in the anterior-posterior (36 ≥ y ≥ 0) as well as the ventral-dorsal plane (z > 4). The size of this ROI amounts to 18,912 mm^3^ (for an illustration see Fig. S1A in the Supplementary Information). Furthermore, to consider other brain regions implicated in the control and monitoring of response tendencies, we also used a binarized mask based from a Neurosynth meta-analysis of 598 human neuroimaging studies associated with cognitive control (retrieved on September 29, 2021 from http://neurosynth.org/analyses/terms/cognitive%20control/) in a supplemental analysis. In addition to the dACC, this mask includes regions in the dorsolateral and ventrolateral prefrontal cortex, the posterior cingulate cortex as well as regions in the parietal lobe. Its size amounts to 11,360 mm^3^ (see Fig. S1B in the Supplementary Information).

On the explorative level, we also tested for general differences in activation as well as for specific differences between the devalued versus the other two Pavlovian cues between the two Transfer Test phases in brain regions that are responsible for the valuation of incoming stimuli or action outcomes (vmPFC) and areas responsible for the implementation of behavior control (amygdala, putamen). With respect to the vmPFC, we used a bilateral mask of area 14 m as described in Mackey and Petrides (2014) by relying on the symmetric population maps binarized using a threshold of 4 (i.e., the resulting ROI mask contains all voxels where the probability exceeds 50% that the respective region is associated with this area). The size of this ROI amounts to 4,452 mm^3^. Bilateral masks for amygdala (3,744 mm^3^) and putamen (16,466 mm^3^) were extracted from the automated anatomical labeling atlas 3 (Rolls et al., 2020). These additional ROIs are illustrated in Figure S2 in the Supplementary Information.

Based on the behavioral results, we furthermore carried out an explorative analysis on the relationship between the extent of behavioral devaluation effects and the differences in neural activation between Transfer Test phases (i.e., before and after devaluation). To this aim, we correlated the differences of the z-scored magnitudes of PIT effects between both Transfer Test phases and the corresponding contrast images (i.e., difference between CS1 vs. CS− between Transfer Test phases). Small volume corrections were applied for the same ROIs as described above.

Throughout the results section, we report peak activation coordinates in MNI space along with *t*- and *p*_FWE_-values. We additionally planned to report effects in brain regions that are not part of the respective masks when reaching statistical significance on a whole-brain level, but this did not happen.

##### 4.2 Results

All statistical tests reported below were preregistered at https://doi.org/10.17605/OSF.IO/K8YGB, unless otherwise noted.

###### 4.2.1 Behavioral data

####### 4.2.1.1 Effect of outcome devaluation

If the devaluation treatment was effective, the rate of button presses working for the now-devalued outcome (R1) during presentations of the neutral cue (CS−) should be lower in the second transfer test (after devaluation) than in the first transfer test (before devaluation). This is indicated by a significant effect of CS− on R1 responding in a univariate ANOVA, which is identical to a paired t-test for R1 responding in Transfer Test 1 (*M* = 9.68, *SD* = 8.9) and Test 2 (*M* = 1.05, *SD* = 2.8). A corresponding one-tailed t-test revealed a large difference, *t*(40) = 5.81, *p* < .001, *d_z_* = 0.908, 95% CI [0.53, 1.26].

In addition to the response rates, an exploratory analysis (not preregistered) examined the ratings of the monetary outcomes (African currencies) using a 3 x 2 rm-ANOVA with the factors *Currency* (1 = devalued, 2, 3) and *Time Period* (before vs. after devaluation). The main effect of *Time Period*, *F*(1, 40) = 5.26, *p* = .027, η_p_^2^ = .116, and the main effect of *Currency*, *F*(1.386, 55.437) = 102.55, *p* < .001, η_p_^2^ = .719, were significant. The interaction term was also significant, *F*(1.32, 53.13) = 79.75, *p* < .001, η_p_^2^ = .666. As expected, ratings did not significantly differ before revaluation and ratings of O1 were significantly lower relative to the others after the devaluation of that currency (see Table 2).

**Table 2.** Ratings of monetary outcomes (M, SD) before and after the devaluation of O1.

Outcome (currency)	Before	After
O1	2.82 (1.02)	1.97 (0.79)
O2	2.85 (1.06)	3.82 (0.86)
O3	2.90 (0.99)	3.87 (0.81)

*Note.* Ratings on a scale from 1 (very bad) to 5 (very good).

####### 4.2.1.2 Specific PIT effect before devaluation (PIT Test 1).

**Fig. 2.**
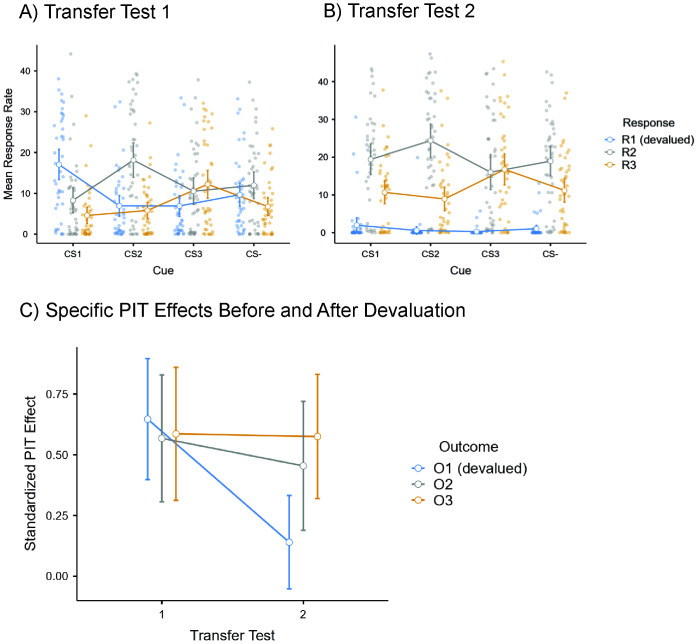
Outcome-selective PIT effects during the first transfer test (Panel A), the second transfer test (Panel B), and compared across both transfer tests (Panel C). *Note.* Error bars show the 0.95 confidence intervals. Dots show individual data points. PIT scores shown in Panel C are z-transformed difference scores (for details see the Analyses of Behavioral Data section). CS = conditioned cue, R = response, O = monetary outcome.

The conditioned cues (CS1, CS2, CS3) should specifically increase the rate of button presses associated with the same outcome (R1, R2, R3, respectively) relative to the baseline condition (CS−). Statistically, this is expressed in a significant 2-way interaction effect between *Cue* (CS1, CS2, CS3, CS−) and *Response* (R1, R2, R3). In the 4 x 3 ANOVA of the button presses in the first transfer test, the main effect of *Cue* was not significant, *F*(1.54, 61.69) = 2.55, *p* = .099, η_p_^2^ = .060. The main effect *Response* was significant, *F*(1.64, 65.93) = 8.43, *p* = .001, η_p_^2^ = .174. The two-way interaction effect was also significant, *F*(1.70, 68.07) = 17.98, *p* < .001, η_p_^2^ = .310, in line with a specific PIT effect (see Panel A in Fig. 2).

####### 4.2.1.3 Specific PIT effect after devaluation (PIT Test 2).

After devaluation of the outcome associated with CS1 and R1, the remaining conditioned cues (CS2, CS3) should still increase working for corresponding intact currencies (R2, R3, respectively) relative to the baseline condition (CS−). Thus, a 3 (CS2, CS3, CS−) x 2 (R2, R3) ANOVA on the response rates in the second transfer test was performed. In this ANOVA, the main effect of *Cue*, *F*(1.332, 53.29) = 5.21, *p* = .018, η_p_^2^ = .115, and the main effect *Response* were significant, *F*(1, 40) = 20.07, *p* < .001, η_p_^2^ = .334. Confirming a specific PIT effect, the two-way interaction effect was also significant, *F*(1.205, 48.18) = 10.50, *p* < .001, η_p_^2^ = .208 (see Panel B in Fig. 2).

####### 4.2.1.4 Comparison of specific PIT effects between transfer tests before and after devaluation.

Specific PIT effects were computed for each currency by subtracting the z-transformed response rate in the baseline condition (CS−) from the response rate during presentations of a CS associated with the same outcome (i.e., CS1:R1-CS−:R1, CS2:R2-CS−:R2, and CS3:R3-CS−:R3). This computation was done for each transfer test. Resulting values were then entered in a repeated-measures ANOVA with *Specific PIT Effect* (O1, O2, O3) and *Transfer Test* (1, 2), whereby O1 designated the (later) devalued currency. Panel C in Figure 2 displays the results.

The main effect of *specific PIT effect* was not significant, *F*(2, 80) = 2.62, *p* = .078, η_p_^2^ = .062, but the main effect of *Transfer Test* was, *F*(1, 40) = 6.92, *p* = .012, η_p_^2^ = .148. Most importantly, the interaction effect was significant, *F*(1.72, 68.78) = 4.51, *p* = .019, η_p_^2^ = .101. Follow-up comparisons of specific PIT effects in both transfer tests using paired *t*-tests confirmed a significant reduction of the specific PIT effect after devaluation of the associated outcome (O1), *t*(40) = 3.82, *p* < .001, *d_z_* = 0.597, 95% CI [0.26, 0.92]. The PIT effect for the devalued outcome did also not differ significantly from zero in a one-sample *t*-test, *t*(40) = 1.46, *p* = .150, *d_z_* = 0.229, 95% CI [-0.08, 0.53]. In contrast, magnitudes of PIT effects associated with the intact currencies were not significantly different in both transfer tests, with *t*(40) = 0.94, *p* = .349, *d_z_* = 0.148, 95% CI [-0.16, 0.45], for O2, and *t*(40) = 0.08, *p* = .934, *d_z_* = 0.13, 95% CI [-0.29, 0.31], for O3.

###### 4.2.2 Functional imaging data

####### 4.2.2.1 Simple contrasts of CSs+ to CS− within each transfer test phase.

To examine activation increases for the conditioned cues (CS1, CS2, CS3) relative to the baseline condition (CS−), we calculated simple contrasts for each cue separately for both Transfer Test Phases. For Transfer Test 1, these analyses revealed significant activation increases in the dACC for the CS1 (10/12/44, *t* = 4.73, *p*_FWE_ = .008) and the CS2 (-6/26/34, *t* = 4.69, *p*_FWE_ = .011) but no such effect for CS3. Moreover, a significant activation of the CS1 versus CS− contrast was observed in the right middle frontal gyrus (44/22/30, *t* = 4.57, *p*_FWE_ = .015)—a region associated with cognitive control (according to a Neurosynth mask). Analogous contrasts for CS2 and CS3 did not show this effect. For Transfer Test 2, no significant differences for any CS relative to CS− were evident.

Additional preregistered analyses examining activity differences in the vmPFC, amygdala, and putamen did not show any significant differences between the conditioned cues (CS1, CS2, CS3) relative to CS− in either transfer test.

####### 4.2.2.2 Comparison of CSs+ relative to CSs− between transfer test phases.

**Fig. 3.**
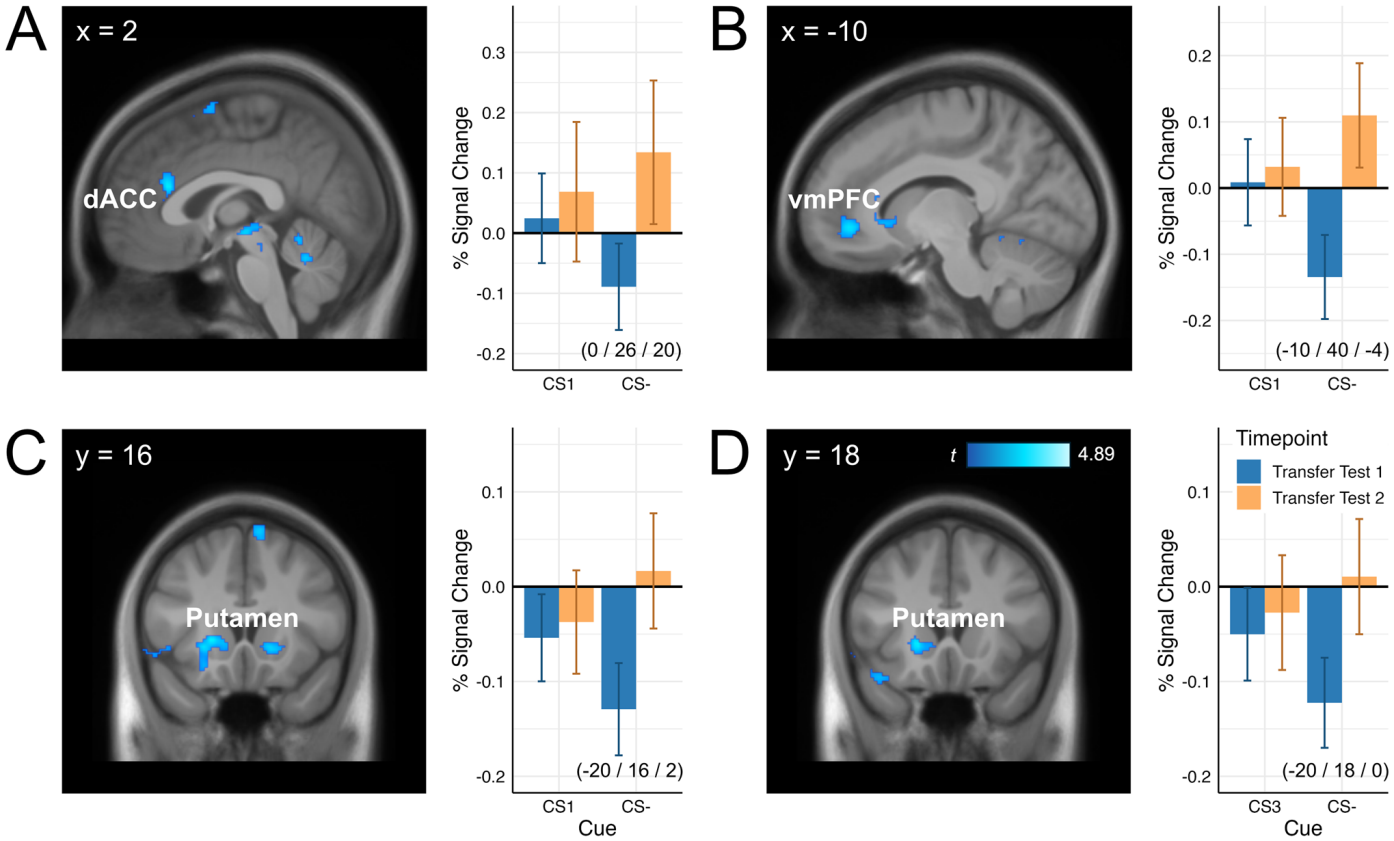
Activation differences of CSs relative to CSs− between transfer test phases. *Note.* Significantly reduced CS1 versus CS− differences in the second as compared to the first Transfer Test phase were observed in the dorsal anterior cingulate cortex (dACC, panel A), the ventromedial prefrontal cortex (vmPFC, panel B), and the putamen (panel C). Comparable effects for CS3 versus CS− were found in the putamen (panel D). On the left side of each panel, contrasts are depicted as t-maps overlaid on an average anatomical image of all participants. For visualization purposes, statistical parametric maps were thresholded at *p* < .005 (uncorrected) at a cluster extent of 100 voxels. On the right side, extracted signal changes are illustrated at the respective peak voxels of the interaction contrast with error bars showing standard errors of the mean.

A comparison of the average activation differences of all Pavlovian cues (CS1, CS2, CS3) minus the CS− in the second, as compared to the first, transfer test did not reveal significant differences in the dACC or in the Neurosynth mask. However, in the preregistered analyses for the additional ROIs, we observed a significantly reduced activation difference in the second transfer test in the putamen (-18/16/0, *t* = 4.89, *p*_FWE_ = .006), but not in the vmPFC or the amygdala. Comparison between Transfer Test phases during CS1 presentations, associated with the devalued outcome, relative to CS− revealed that activation of the dACC (0/26/20, *t* = 4.73, *p*_FWE_ = .008), the vmPFC (-10/40/-4, *t* = 4.11, *p*_FWE_ = .023), and the putamen (-20/16/2, *t* = 4.53, *p*_FWE_ = .017) was significantly reduced in the second transfer test compared to the first (see Fig. 3). No significant differences for this contrast were observed in the amygdala or the Neurosynth mask apart from the dACC. Comparable analyses for the contrasts involving CS2 and CS3 only showed a significantly reduced activation in the second Transfer Test phase in the putamen for the CS3-CS− contrast (-20/18/0, *t* = 4.27, *p*_FWE_ = .026). No further significant differences were observed in any of the ROIs.

####### 4.2.2.3 Correlations between behavioral and neural devaluation effects.

A non-preregistered correlative analysis, examining the relationship between the extent of behavioral devaluation effects and differences in neural activation between the Transfer Test phases, did not reveal any significant effects in any ROI.

#### 5 Discussion

Based on the EVC theory (Shenhav et al., 2016), we hypothesized that cognitive control would be intensified in the outcome-selective PIT paradigm when the costs of obtaining a devalued outcome outweighs the costs of suppressing the cue-motivated response. At the neural level, we therefore expected *increased* activity in the dACC during presentations of the CS associated with the devalued outcome relative to the baseline (CS−). However, neuroimaging data showed the opposite result: dACC activity *decreased* after outcome devaluation in the second transfer test relative to the first transfer test. This finding suggests that devaluation of the action outcome did not enhance cognitive control over cue-instigated response tendencies.

Notably, activity in the dACC was significantly increased when participants responded to CSs relative to the CS− during the first transfer test, before the devaluation of one of the outcomes. This increase in activation could reflect enhanced motivation to work for the monetary rewards (Holroyd & Yeung, 2012; Rushworth & Behrens, 2008). However, significantly increased dACC activity was only observed for CS1 and CS2, and not for CS3. A post hoc explanation for this difference could be that CS3 shared the outcome with a button press that was the least accessible on the response pad, requiring a press with the ring finger, and that was less preferred compared to the others (see Fig. 2A). This ergonomic difference may explain why dACC activity was not significantly increased for all three CS+ stimuli equally relative to the CS−.

Devaluation of one of the monetary outcomes had the expected effect on the participants’ behavior, as they largely stopped working for that outcome in the second transfer test phase (see the flat blue line in Fig. 2, Panel B). However, dACC activation during this phase did not differ between the devalued CS1 and the CS−. The absence of a difference in neural activation could reflect a functional equivalence of these cues in this phase, as both served as signals of no reward in the second transfer test phase. For example, it is possible that participants learned to ignore the CS1 during the second transfer test phase because of the loss of expected reward. Alternatively, it is possible that the absence of dACC activation reflects successful goal-directed suppression. This explanation would require that other cognitive-control regions than those examined in the present study implemented this suppression, as we did not observe activity differences during CS1 and CS− presentations in regions associated with cognitive control as specified by a Neurosynth-based mask. Note that either of these explanations would imply that the changed value of the outcome associated with CS1 was accessible during, or at least at the beginning of, the second transfer test.

Comparisons between the transfer test phases revealed significant differences in the activation of the dACC, the vmPFC, and the putamen during presentations of the (devalued) CS1 relative to the CS−. First, it is important to note that the largest signal change between test phases was observed for presentations of the CS−, which was selected as the baseline for our comparisons (see Fig. 3). This fluctuation in signal strength is difficult to explain, given that the function of the CS− remained unchanged across phases. However, the introduction of a secondary signal of no reward—the CS1 following the instructed devaluation—may have increased the difficulty of discriminating the CS− from the other cues in the second transfer test phase compared to the first. This may post hoc explain the increase in dACC activity during CS− presentations in the second transfer test phase compared to the first. Furthermore, activity in the dACC and in cognitive-control regions identified using the Neurosynth-based mask did not differ across test phases when all three CS+ were contrasted with the CS−. This finding does not support explanations that attribute the observed CS− signal change to a general difference between transfer test phases, such as a difference in task engagement or in motivational arousal.

Notably, the increase in dACC activation between transfer test phases for the CS− was significant only in comparison with the devalued CS1, and not in comparison with the non-devalued cues (CS2 and CS3). Thus, the devaluation of CS1 reduced activity in the dACC relative to the selected baseline (CS−) in the second transfer test compared to the first one. To be clear, we do not have an explanation for this relative decrease across the test phases. This finding is also not specific for the selected baseline CS−, as activity in dACC was similarly reduced when the green 2-s blank period presented before the cue was used as the baseline for comparisons (see Fig. S3 in the Supplementary Information). Nevertheless, the decrease clearly runs counter to the hypothesized *increase* in dACC activation due to effortful inhibition of a cue-dependent response tendency working for the devalued outcome.

In addition to the dACC, activity in the vmPFC and in the putamen was also reduced for the contrast between the CS1 and the CS− after, compared to before, devaluation. The vmPFC is a major hub for guiding value-based decisions and is involved in the revaluation of rewards based on changing contexts or new information (Gläscher et al., 2009; Kroker et al., 2024). Human neuroimaging studies have demonstrated that the vmPFC is specifically involved in processing the receipt of monetary rewards: upon receiving an expected reward, activation increases, whereas it decreases when an expected reward is not received (Knutson et al., 2001, 2003). In the present study, the reduced vmPFC activity in the second transfer test may reflect the updating of the previously rewarded action due to the receipt of no reward in this phase. However, this process must have relied on an internal representation of non-reward, as rewards were generally not delivered during the transfer test due to the formal extinction procedure. We consider this plausible, given that participants were explicitly informed that they could still earn rewards during the transfer phases, although their earnings were not displayed on the screen.

The putamen is primarily implicated in habit formation and is also involved in updating rewarded stimulus-response associations, particularly when motor skills or habits need to be modified (Balleine et al., 2007; Lipton et al., 2019). O’Doherty et al. (2004) proposed complementary roles for the ventral and dorsal parts of the striatum, with the ventral striatum (nucleus accumbens) serving as a “critic” that learns to predict future rewards and the dorsal striatum (putamen and caudate nucleus) serving as an “actor” that maintains and updates information about the rewarding outcomes of actions. The reduced activation of the putamen in the second transfer test during presentations of the devalued CS1 relative to the CS− compared to the same contrast prior to devaluation could be related to policy updating following the instructed outcome devaluation. This may reflect a shift whereby actions associated with no long-term reward were selected less frequently on subsequent trials.

In addition, activity in the putamen was generally reduced in the second transfer test phase following outcome devaluation compared to the first test, collapsed across all CSs relative to the CS−. This activation difference could reflect the acquisition of sensorimotor associations, or habit formation, during the first transfer test. It is important to note that research on the putamen’s role in habitual behavior has produced mixed findings regarding its activity levels after habit formation. While some studies report increased putamen activity as behaviors become habitual (e.g., Tricomi et al., 2009), others found decreased activity in anterior portions of the caudate nucleus—implicated in value-based action selection—and no significant difference in putamen activity after extensive training (Choi et al., 2020; Gera et al., 2023). Research also demonstrated that early in learning, activity is greater in associative areas of the anterior putamen, whereas after habit formation, activity in this subregion declines and is shifted to posterior sensorimotor areas of the putamen (Guida et al., 2022; Lehéricy et al., 2005; Patterson & Knowlton, 2018). Future research may focus on particular subregions of the dorsal striatum to investigate its dynamic functional reorganization when responding to cues during a PIT task.

At the behavioral level, the results of the present study closely replicated previous findings using similar procedures with monetary incentives (Allman et al., 2010; Eder & Dignath, 2016a) or biological reinforcers (Eder & Dignath, 2016b). The adjustment of behavior after outcome devaluation, which eliminated the cue-instigated response tendency observed in the first phase while maintaining those working for the valued outcomes, clearly demonstrates that the current value of the action outcome was accessible during the transfer test, challenging a strict dual controller theory (Dickinson, 1985). Our neuroimaging data, which demonstrate activation differences between test phases in brain centers implicated in reward processing, further support this conclusion. However, activation differences were only observed in some reward-related structures (vmPFC) and not in others (amygdala). Additional research is needed to clarify the reward-related processes and how they are mapped onto neural activations following the outcome devaluation in the PIT task.

The present research aimed to examine whether cognitive control is allocated to cue-dependent action tendencies that produce no reward after devaluation of the associated outcome. Our clear answer to this question is: it is not! Brain regions implicated in cognitive control, most notably the dACC, were less active after the devaluation of the cued outcome compared to before. This conclusion is, of course, preliminary, as it is limited by the specifics of the employed PIT paradigm, the use of symbolic monetary rewards, and the reliance on instructed devaluation. For example, it is possible that controlling the keypress rate in the presence of the cues was effortless and therefore did not require cognitive control. In this case, however, PIT theories that attribute outcome-selective transfer effects in this type of tasks to automatic (habitual) response tendencies, which are suppressed by goal-directed processes to maximize reward, would also need to be revised.

## Supplementary Material

Supplementary Material

## Data Availability

A report of the pilot study and the associated raw data can be accessed at https://osf.io/79gew/. The raw data underlying the behavioral findings reported in this manuscript and a syntax file used for data analysis were deposited at https://osf.io/2dspv/. The neuroimaging data are not publicly available due to data protection regulations but can be shared upon personal request with eligible researchers.

## Footnotes

* We also conducted an exploratory analysis in which regressors for individual responses were not included. This approach yielded results that were largely comparable.
