# Supplementary material for: Motivational control of habits: A preregistered fMRI study
Source: Imaging Neurosci (Camb). 2025 Aug 7;3:IMAG.a.100. doi: 10.1162/IMAG.a.100 (PMC12336060; doi:10.1162/IMAG.a.100)
Supplement: Supplementary Material [file IMAG.a.100_supp.pdf]

Supplementary Material

to “Motivational Control of Habits: A Preregistered fMRI Study”

Additional Details on MRI Analyses and Results

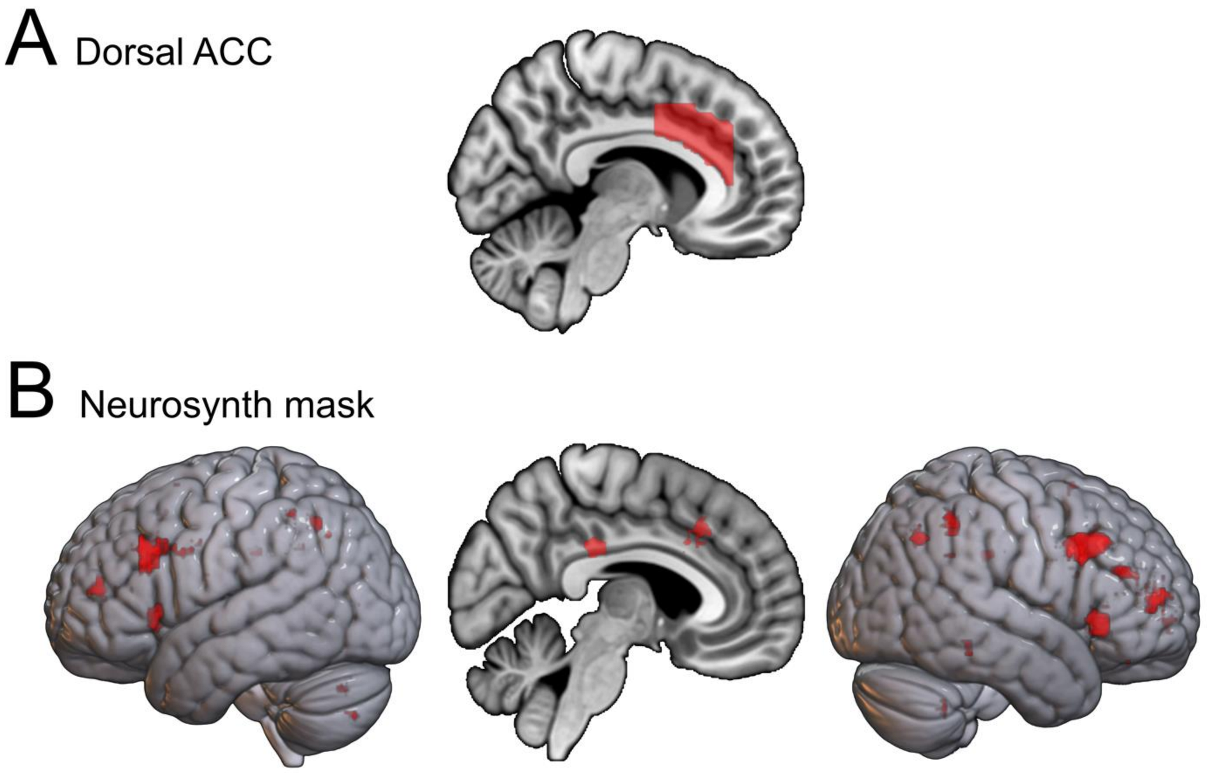

Figure S1: Illustration of the regions of interest (ROIs) used in the primary analyses. A) Dorsal anterior cingulate (dACC) ROI. B) Binarized mask of a Neurosynth meta-analysis of human neuroimaging studies associated with cognitive control.

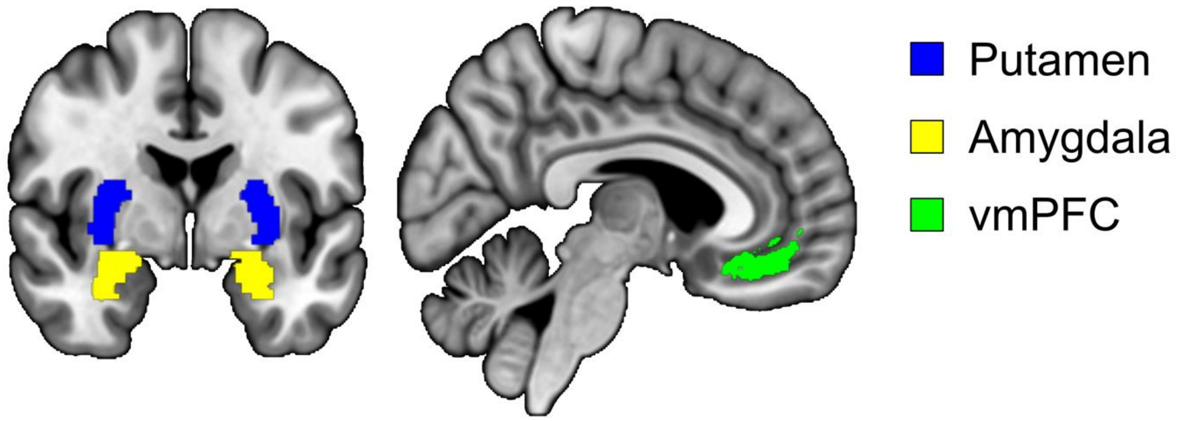

Figure S2: Illustration of the regions of interest (ROIs) used in further explorative analyses encompassing the bilateral putamen and amygdala as well as the ventromedial prefrontal cortex (vmPFC).

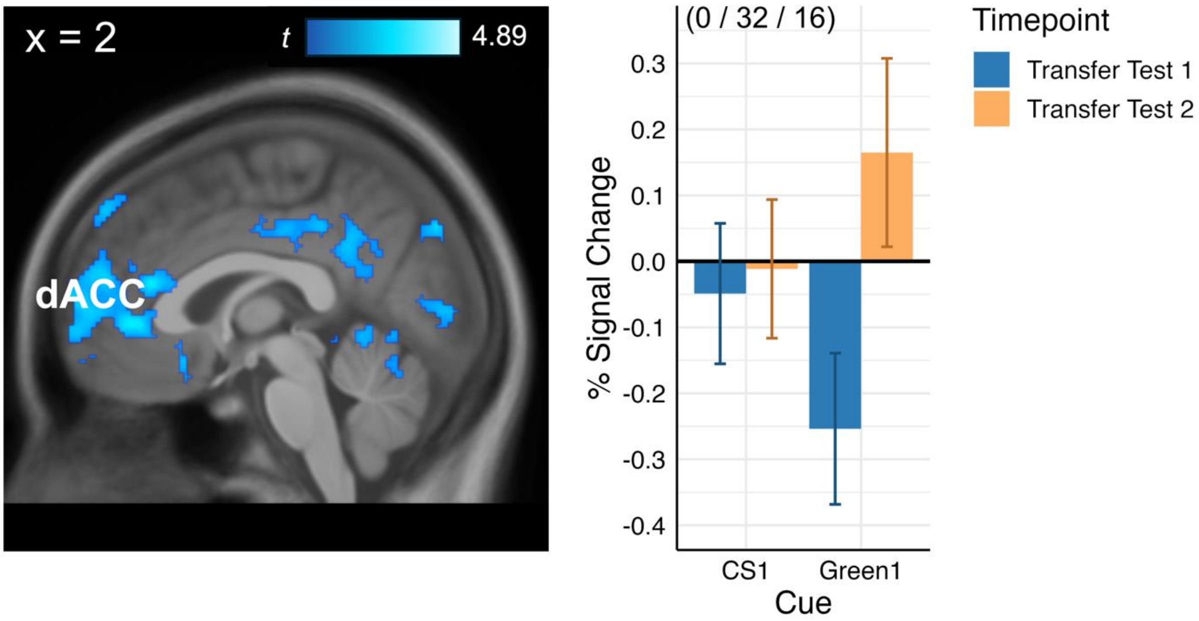

Figure S3: Activation differences of the CS1 relative to the green 2-second blank period presented before the cues between transfer test phases. The analysis revealed a significant interaction effect in the dorsal anterior cingulate cortex (dACC, 0/32/16, t = 4.44, p_{FWE} = .021). On the left side, the interaction contrast is depicted as a t-map overlaid on an average anatomical image of all participants. For visualization purposes, the statistical parametric map was thresholded at p < .005 (uncorrected) at a cluster extent of 100 voxels. On the right side, extracted signal changes are illustrated at the respective peak voxel of the interaction contrast with error bars showing standard errors of the mean.

Table S1

Summary of the Research Plan and Main Results

Question	Hypothesis	Sampling plan	Analysis Plan	Rationale for deciding the sensitivity of the test for confirming or disconfirming the hypothesis	Interpretation given different outcomes	Theory that could be shown wrong by the outcomes	Results
Is the insensitivity of cue-motivated action tendencies to posttraining changes in reward values an intrinsic design feature of habits (as proposed by dual-action psychologies) OR is it because the benefits of controlling "habitual" action	According to EVC, cognitive control is intensified when the benefits of suppressing a dominant action tendency will justify the intrinsic costs of engaging in control. A central hub for these calculations on the neural level is the dorsal anterior cingulate cortex (dACC). In a Pavlovian-to-instrumental (PIT) transfer test, dACC activity	*N* = 41 A-priori power analysis for the detection of an increased dACC activation after relative to before the outcome devaluation in a one-tailed paired t-test with	GLM approach with Pavlovian Cue and Responses as regressors for neural activations in PIT tests before and after the devaluation treatment; follow-up analyses with t-test comparisons of activation differences before and after devaluation of the associated	ES estimates obtained from a behavioural pilot study (d_z = 0.55) and from a source study (Eder & Dignath, 2016b, Exp 2, d_z = 0.53). ES = mean difference in the magnitudes of behavioural PIT effects before and after outcome devaluation.	Increased dACC activity after outcome devaluation would be in line with the EVC model of dACC function in habit control. Finding no dACC effect, and/or observing activation differences in unrelated brain regions, would not	Increased dACC activity after devaluation of the outcome would support EVC theory and challenge dual action psychologies that claim a structural independence of habits from outcome representations.	Not supported: dACC activity was decreased after outcome devaluation

tendencies do not outweigh the intrinsic costs of engaging in control (as suggested by expected value of control theory, EVC)?	should increase during presentations of Pavlovian cues associated with devalued outcomes relative to cues associated with non-devalued/neutral outcomes and in comparisons with PIT tests performed before the devaluation.	1-β = 0.95 and α = .05.	outcome (for details see Specification of fMRI Models for a Test of Brain Activity Hypotheses)		support this model.		
Was the devaluation treatment effective?	Working (response rate) for the devalued outcome O1 should be lower in Transfer Test 2 after compared to before devaluation in Transfer Test 1	*N* = 41	ES = difference between response rates (R1) in the first and second transfer tests. (1) Comparison in a paired t-test. (2) If no significant test result in (1), then TOST procedure for the rejection of SESOI d_z = 0.40.	ES in the pilot study: d_z = 1.1 (95%CI [0.58, 1.61]). Sufficient power (0.80) for the detection of *d* ≥ 0.40 in one-sample t-tests (TOST).	Significant test result in (1) would confirm that the devaluation treatment was effective. Non-significant TOST result in (2) would indicate absence of a meaningful effect (= failure).	Manipulation check (behavioural data). Failure would threaten the conclusiveness of the study: Report is published at OSF but will not be submitted to PCI for Stage 2 approval.	Supported

Was the procedure appropriate for generating cue-dependent ('habitual') action tendencies (Transfer Test 1)?	Pavlovian cues (CS1, CS2, CS3) should specifically increase numbers of keypresses that were associated with the same outcome (R1, R2, R3) relative to the baseline condition (with presentations of CS−).	*N* = 41	ES = specific PIT effect calculated for Transfer Test 1 (1) 2-way interaction effect between Pavlovian Cue and Instrumental Relation in a 4x3 rm-ANOVA (2) If no significant test result in (1), then TOST procedure for the rejection of SESOI d_z = 0.40.	ES in the pilot study: η_p^2 = 0.238 (d_z = 0.85, 95%CI [0.37, 1.31]). Sufficient power (0.80) for the detection of $d \geq 0.40$ in one-sample t-tests (TOST).	Significant test result in (1) would confirm cue-dependent response tendencies in Test 1. Non-significant TOST result in (2) would indicate absence of a meaningful effect (= failure).	Manipulation check (behavioural data). Failure would threaten the conclusiveness of the results. Report is published at OSF but will not be submitted to PCI for Stage 2 approval.	Supported
Was the procedure appropriate for generating cue-dependent ('habitual') action	Non-devalued Pavlovian cues (CS2, CS3) should specifically increase numbers of keypresses associated with	*N* = 41	ES = specific PIT effect (R2, R3), calculated for Transfer Test 2 (1) 2-way interaction effect	ES in the pilot study: η_p^2 = 0.43 (d_z = 0.94, 95%CI [0.45, 1.42]). Sufficient power (0.80)	Significant test result in (1) would confirm cue-dependent response	Manipulation check (behavioural data). Failure would seriously question the	Supported

tendencies (Transfer Test 2)?	the same outcome (R2, R3) relative to the baseline condition (with presentations of CS−).		between Pavlovian Cue and Instrumental Relation in a 3x2 rm-ANOVA (2) If no significant test result in (1), then TOST procedure for the rejection of SESOI d_z = 0.40.	for the detection of *d* ≥ 0.40 in one-sample t-tests (TOST).	tendencies in Test 2. Non-significant TOST result in (2) would indicate absence of a meaningful effect (= failure).	conclusiveness of the results. Report is published at OSF but will not be submitted for Stage 2 approval to PCI.	

Note. ES = effect size; SESOI = minimum effect size of interest; TOST = two one-sided tests procedure; OSF = Open Science Framework. The Preregistered Stage 1 protocol, which included this table without knowledge of the results, is available at https://osf.io/k8ygb (date of in-principle acceptance: 08-02-2022)
